# Monocytes engineered with iSNAP inhibit human B‐lymphoma progression

**DOI:** 10.1002/btm2.10285

**Published:** 2022-01-12

**Authors:** Haohsiang Wu, Siamak Amirfakhri, Hsin‐Hung Lin, Hannah Hollandsworth, Filemoni Filemoni, Yahan Liu, Yiqian Wu, Julie Y. S. Li, Hongquan Xu, Shu Chien, Michael Bouvet, Yingxiao Wang

**Affiliations:** ^1^ Department of Bioengineering and Institute of Engineering in Medicine University of California San Diego San Diego California USA; ^2^ Institute of Clinical Medicine, Stem Cell Research Center National Yang Ming University Taipei Taiwan; ^3^ Department of Surgery, Moores Cancer Center University of California, San Diego San Diego California USA; ^4^ Department of Surgery VA San Diego Healthcare System San Diego California USA; ^5^ Department of Statistics University of California Los Angeles California USA; ^6^ Department of Medicine University of California San Diego San Diego California USA

**Keywords:** cancer immunotherapy, CD47, Don't eat me signal, engineering monocytes, macrophage polarization

## Abstract

Monocytes are important regulators for the maintenance of homeostasis in innate and adaptive immune system and have been reported to play important role in cancer progression. CD47‐SIRPα recognition is a coinhibitory immune signal to inhibit phagocytosis in monocytes and macrophages and has been well‐known as the “Don't eat me” signal. By using an approach of integrated sensing and activating proteins (iSNAPs), we have rewired the CD47‐SIRPα axis to create iSNAP‐M which activates pathways in engineered human monocytes (iSNAP‐MC). The mRNA expression levels of the monocyte/macrophage markers CD11b, CD14, and CD31 are upregulated in iSNAP‐monocytes (iSNAP‐MC). With PMA induction, the iSNAP‐MC‐derived macrophages (iSNAP‐MΦ) showed upregelation in CD86 and CD80, but not CD206. TNFα expression and secretion were also increased in iSNAP‐MΦ. Furthermore, the injection of iSNAP‐MC into mice bearing human B‐lymphoma tumors led to the suppression of tumor progression. Therefore, the engineered monocytes, via blockage of coinhibitory immune signals by rewiring CD47‐SIRPα axis, can be applied to suppress target tumors for cancer immunotherapy.

## 1 INTRODUCTION

Human innate and adaptive immune system maintains homeostasis and prevents carcinogenesis. Innate immunity also plays a crucial role for the activation of adaptive immunity. Monocytes are innate immune cells which can migrate into tissues and differentiate into macrophages in response to inflammatory stimulations and chemotaxis.^1^ Macrophages are phagocytosis cells and present antigens to activate T cells through the binding of major histocompatibility complex II and T‐cell receptors. Macrophages can also engulf tumor cells by phagocytosis and further activate T cell response via antigen presenting.^2^ Signal regulatory protein α (SIRPα) is a membrane glycoprotein highly expressed on macrophages, serving as a coinhibitory signal that interacts with CD47 and inhibits phagocytosis; so called the “Don't eat me” signal.^3^ SHP1 and SHP2 are downstream tyrosine phosphatases of SIRPα activation that are involved in multiple signaling pathways to modulate macrophage phenotype.^4^, ^5^ Systemic injection of macrophages coated by antibodies for tumor targeting and for SIRPα blocking can repress tumor growth.^6^ Therefore, the CD47‐SIRPα axis in macrophages is considered as a crucial target for antitumor immunotherapy.

When monocytes are differentiated to macrophages inside tissues, they are further activated. Polarization of activated macrophages into proinflammatory state (M1) and anti‐inflammatory state (M2) has been reported to occur via different cytokine stimulations in vitro.^7^ M1 macrophages secrete TNFα, IL‐6, IL‐8 and activate Th1 response, whereas M2 macrophages secrete IL‐10 and activate Th2/Th17 response. In vivo, macrophages have more diverse and plastic responses to changes in local microenvironment.^8^ Tumor‐associated macrophages (TAMs) have been reported to promote tumor progression and develop M2 polarization in local tumor microenvironment.^9^, ^10^ In fact, a high degree of TAM infiltration has been reported to correlate with poor diagnosis in clinical studies. Consistent with this finding, switching M2 to M1 phenotype of TAMs can inhibit tumor growth.^11^ Therefore, manipulating macrophage phenotypes in tumors can be an appealing therapeutic strategy for solid tumors.

In the last few decades, engineered macrophages with the blockage of CD 47‐SIRPα recognition have shown promising efficacy to reduce tumor growth in non‐Hodgkin lymphoma (NHL), acute lymphoblastic leukemia, acute myeloid leukemia, and myeloma.^12^ Previously, we engineered integrated sensing and activating proteins (iSNAPs) that are capable of rewiring the signaling of the inhibitory SIRPα proteins into activating pathways in macrophages.^13^ Overexpressing this iSNAPs in RAW264.7 macrophage cell lines and primary bone marrow‐derived macrophages^13^ enhanced the phagocytic ability of these engineered macrophages. In this study, we introduced the engineered iSNAP in human THP1 monocytes to generate iSNAPs derived monocytes (iSNAP‐MC) and macrophages (iSNAP‐MΦ) and demonstrated that the engineered iSNAP‐MC/MΦ have enhanced efficacy in suppressing human B‐lymphoma in vivo.

## 2 MATERIALS AND METHODS

### 2.1 Cell culture

THP1, iSNAP‐MC, and Toledo cells were cultured in RPMI 1640 (Cat. no. 11875‐093; Invitrogen) supplemented with 10% FBS (Cat. no. 10437028; Thermo Fisher Scientific) and 1% penicillin–streptomycin (Cat. no. 15140‐122; Gibco). The cells were subcultured, and fresh culture medium was changed twice in a week. To induce differentiation into macrophages, THP1 and iSNAP‐MC were treated with 20 ng/ml phorbol 12‐myristate 13‐acetate (PMA; Abcam) for 2 days.

### 2.2 The establishment of iSNAP‐MC

The genetic construct of iSNAP‐M was described in our previous study^13^ and introduced into THP1 via lentivirus. Cells were then sorted by the expressed YFP fluorescence to obtain the iSNAP‐MC. The cells were thereafter washed twice by phosphate‐buffered saline (PBS) and cultured in RPMI 1640 supplemented with 10% FBS and 1% penicillin–streptomycin.

### 2.3 Quantitative PCR

Cells were lysed by TRIzol (Cat. no. 15596‐018; Thermo Fisher Scientific), and then total RNA was extracted by using Direct‐zol™ RNA MiniPrep (Cat. no. R2052; Zymo Research). RNA was quantified and reverse‐transcribed to cDNA by M‐MLV Reverse Transcriptase (Cat. no. 28025013; Thermo Fisher Scientific). Quantitative PCR was done by using SyBr Green Master Mix (Cat. no. 170‐8882; Bio‐Rad), and primers are listed in Table S1. All values were normalized with the human housekeeping gene GAPDH and expressed as mean ± *SD*. Statistical analysis was performed by double tailed *t*‐test. Significance was determined from *p* < 0.05.

### 2.4 Flow cytometry

The cells were collected and washed with PBS (Sigma) twice. Then, the cells were suspended in 100 μl PBS and stained with human CD86 (BD Pharmingen) and CD206 (BD Pharmingen) for 1 h on ice. After PBS washing twice, the cell surface markers were analyzed by flow cytometry (BD Accuri™ C6 Plus).

### 2.5 Cytokine array and ELISA assay

iSNAP‐MC cells were induced to differentiate into macrophages for 48 h by the treatment of 20 ng/m PMA (ab120297; Abcam). The cell conditioned medium was collected and centrifuged to remove cell debris at 1000*g* for 5 min. The supernatant was collected and stored in −80°C. Cell secretion profile was analyzed with a cytokine array (ARY005B; R&D). Secretions of TNFα and IL‐10 in the conditioned medium were quantified by using ELISA kits (DY210‐05 and DY217B‐05; BD Pharmingen). All values are expressed as mean ± *SD* and analyzed statistically by the double tailed *t*‐test. Significance was determined from *p* < 0.05.

### 2.6 Phagocytic ability assay

THP1 and iSNAPs (2 × 10^6^) were differentiated to macrophages by PMA treatment (20 ng/ml) for 48 h. Toledo cells were labeled by Mito Tracker Deep Red (Cat. no. M22426; Thermo Fisher Scientific) for 15 min and washed twice by PBS. The labeled Toledo cells were then pretreated with 10 μg/m; human CD20 antibody (MAB9575; R&D) at 37°C for 1 h and then washed by PBS once. THP1 or iSNAP‐MC derived macrophages were then cocultured with the labeled Toledo cells (5 × 10^6^) for 4 h. After coculturing, the cells were washed twice by PBS and trypsinized for 5 min. The cells were spin down and resuspended for further analysis by flow cytometry.

### 2.7 Animals

Female and male nude mice age 4–6 weeks were purchased from Jackson Laboratories. The animals were housed in a biosafety vivarium and fed an autoclaved laboratory diet. At the conclusion of the study, the mice were euthanized with CO_2_ inhalation, which was confirmed with cervical dislocation. All animal experiments were approved by Institutional Animal Care and Utilization Committee of the UCSD Institutional Animal Care and Use Committee (IACUC S14009).

### 2.8 Tumor establishment

Non‐Hodgkin B‐lymphoma Toledo cell line was purchased from American Type Culture Collection. Toledo cells were infected with *pHIV‐Luc‐ZsGreen* and cultured in RPMI medium 1640 with l‐glutamine, containing 10% FBS and 1% penicillin–streptomycin. After washing three times with cold PBS, the cells (1 × 10^6^) were suspended in a mixture of Matrigel Matrix (CB40234; Corning Life Sciences) and ice‐cold PBS, and injected into the bilateral flanks of nude mice. After 30 days of Toledo cell inoculation, subcutaneous tumors were generated to reach 5 mm in diameter for further experiments.

### 2.9 Treatment plan

The mice were randomized into three groups: No‐treatment, and injections of THP‐1 or iSNAP‐MC. The “No‐treatment” control mice were administered intratumorally with PBS. Mice receiving treatments were administered with THP‐1 or iSNAP‐MC cells (1 × 10^6^) via intratumoral injection. Tumor sizes were measured with a caliper weekly for 3 weeks. IVIS imaging was performed after intraperitoneal injection of d‐luciferin (LUCK‐1G; Gold Biotechnology) and acquired by using a Xenogen IVIS 200 system. Tumor volume was calculated with the formula of volume = (width^2^ × length)/2.^14^

### 2.10 Immunostaining

The tumors were collected and fixed with 4% paraformaldehyde. After dehydration and paraffin embedding, tumor samples were sectioned for immunostaining. Anti‐YFP antibody (MBS833304; MyBioSource) was used to detect injected iSNAP‐MC in the tumors.

### 2.11 Human IL‐1β ELISA

Blood sampling in mice was performed after 1 week of the iSNAP‐MC treatment. After sitting for 30 min in room temperature, blood samples were spin down at 2000*g* for 10 min. Then, the serum was collected and stored in −80°C. Human IL‐1Β in the serum was quantified by using ELISA kits (Thermo Fisher Scientific).

### 2.12 Statistical analysis of tumor growth

We modeled the tumor growth by the 2/3 power law, which assumes that the tumor growth occurs at the surface of a three dimensional solid tumor.^15^ The tumor growth rate at time *t* was computed as (*V*(*t*)^(1/3) − *V*(0)^(1/3))/(1/3), where *V*(*t*) is tumor volume at time *t* and *V*(0) the tumor volume at time 0 (before treatment). We performed regression analysis on tumor growth rates for each week separately, followed by residual analysis for checking model assumptions. Specifically, for each week we built a linear regression model using the tumor growth rates as the responses and three indicator variables representing the three treatment groups as predictors. Initial analysis with all animals (*n* = 16) identified an outlier at Week 3 from the THP1 group. We removed the outlier and repeated the analysis with *n* = 15 animals. Statistical tests were conducted using two‐sided *t*‐tests and *p* values for comparison between groups. Residual analysis on the final model confirmed the accuracy of the model assumptions. Statistical analysis was performed using R (https://www.r-project.org/), a free software environment for statistical computing and graphics.

## 3 RESULTS

### 3.1 Establishment of iSNAP‐MC

**FIGURE 1:**
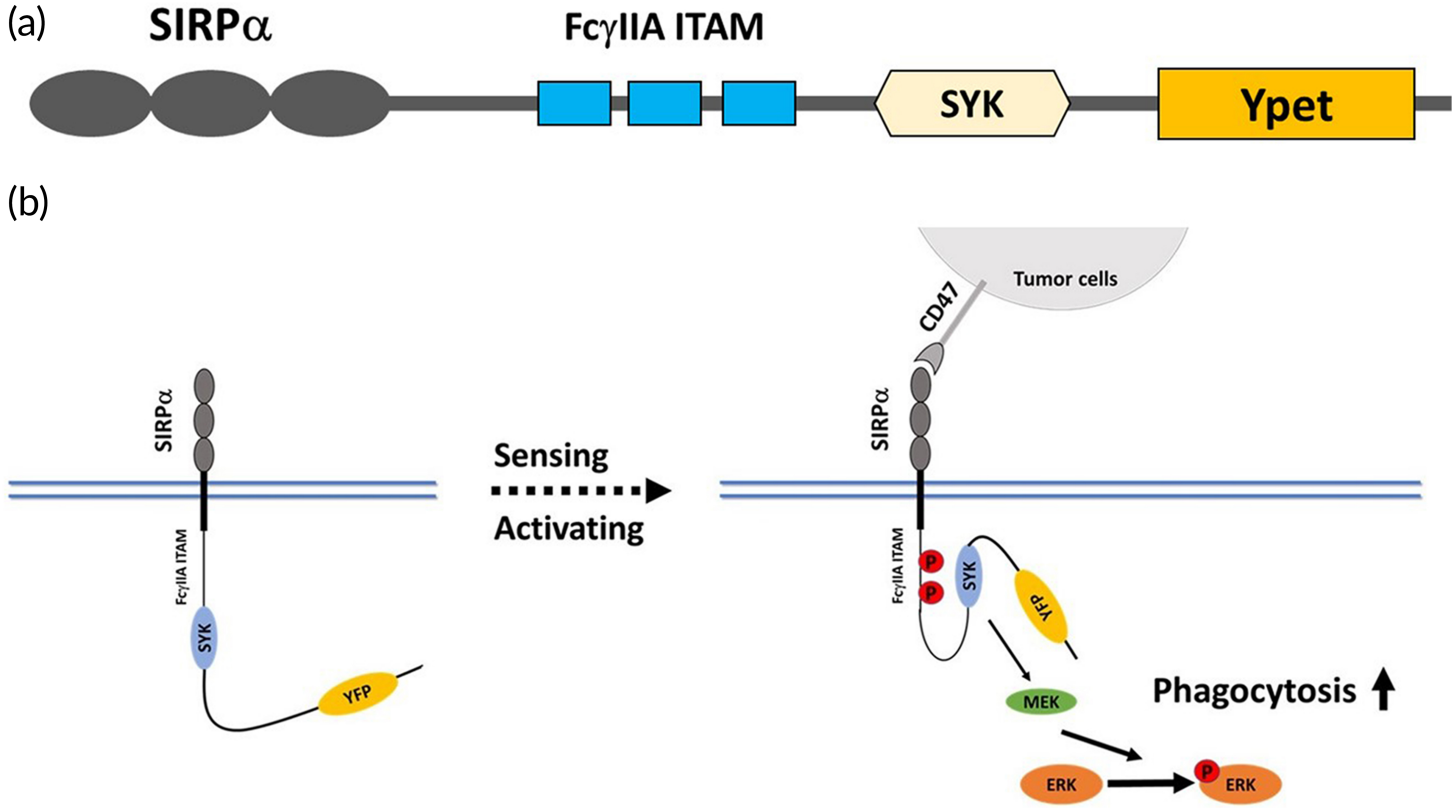
Schematic diagram of engineered monocytes. (a) An approach of integrated sensing and activating proteins (iSNAPs) is used to overexpress engineered SIRPα in THP1 monocytes (iSNAP‐MC). (b) The CD47‐SIRPα axis in iSNAP‐MC is rewired to promote phagocytosis via Erk activation for eradication of cancer cells

By using an approach of iSNAPs,^13^ the intracellular domain of human SIRPα was replaced by a fusion protein comprised of immunoreceptor tyrosine‐based activation motif of Fc‐gamma receptor IIA (FcγR IIA ITAM), SYK kinase, and Ypet (a variant of yellow fluorescent protein, YFP), as shown in Figure 1a. This chimeric protein designed for macrophages is named as iSNAP‐M, with YPet (YFP) as a tag to visualize the iSNAP expression. We introduced the iSNAP in human THP1 monocytes (iSNAP‐MC) to rewire CD47‐SIRPα signaling and promote phagocytotic ability of the iSNAP‐MC derived iSNAP‐MΦ for cancer cell eradication (Figure 1b).

### 3.2 iSNAP‐MC modulates the expression of monocyte/macrophage markers

**FIGURE 2:**
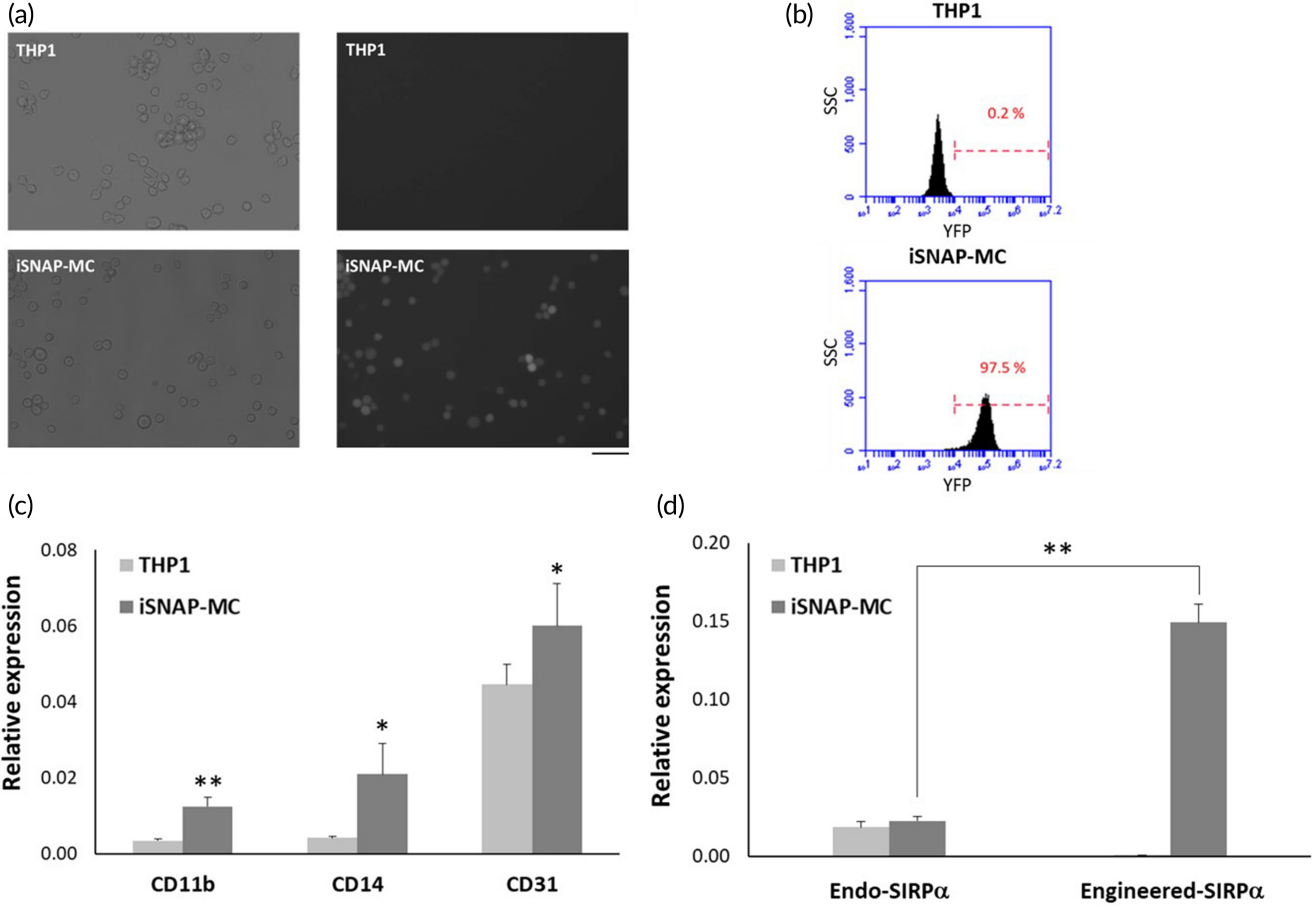
Upregulation of monocyte/macrophage markers in iSNAP‐MC. (a) Engineered SIRPα is overexpressed in iSNAP‐MC. (b) The expression of engineered SIRPα in iSNAP‐MC is quantified by flow cytometry. (c) Expression of monocyte/macrophage markers is upregulated in iSNAP‐MC. (C) mRNA expression levels of endogenous and engineered SIRPα in iSNAP‐MC are quantified by quantitative PCR. Scale bar = 100 μm

As shown in Figure 2a, YFP fused to iSNAP‐M can be clearly detected in iSNAP‐MC, but not in the parental THP1 cells. To confirm the efficiency of iSNAP‐M expression in iSNAP‐MC, YFP^+^ cells were quantified by flow cytometry, and the results showed that over 97% of the iSNAP‐MC are YFP^+^ cells (Figure 2b). The mRNA levels of endogenous and engineered iSNAP‐MC were further quantified by quantitative PCR. The mRNA expression demonstrated that iSNAP‐MCs have upregulated CD11b, CD14, and CD31 (Figure 2c), the monocyte/macrophage markers involved in phagocytosis and leukocyte transmigration.^16^, ^17^, ^18^ The result further showed that the expression of iSNAP‐M in iSNAP‐MC is at levels markedly higher than that of the endogenous SIRPα (Figure 2d).

### 3.3 iSNAP‐MC‐derived macrophages exhibit M1 phenotype

**FIGURE 3:**
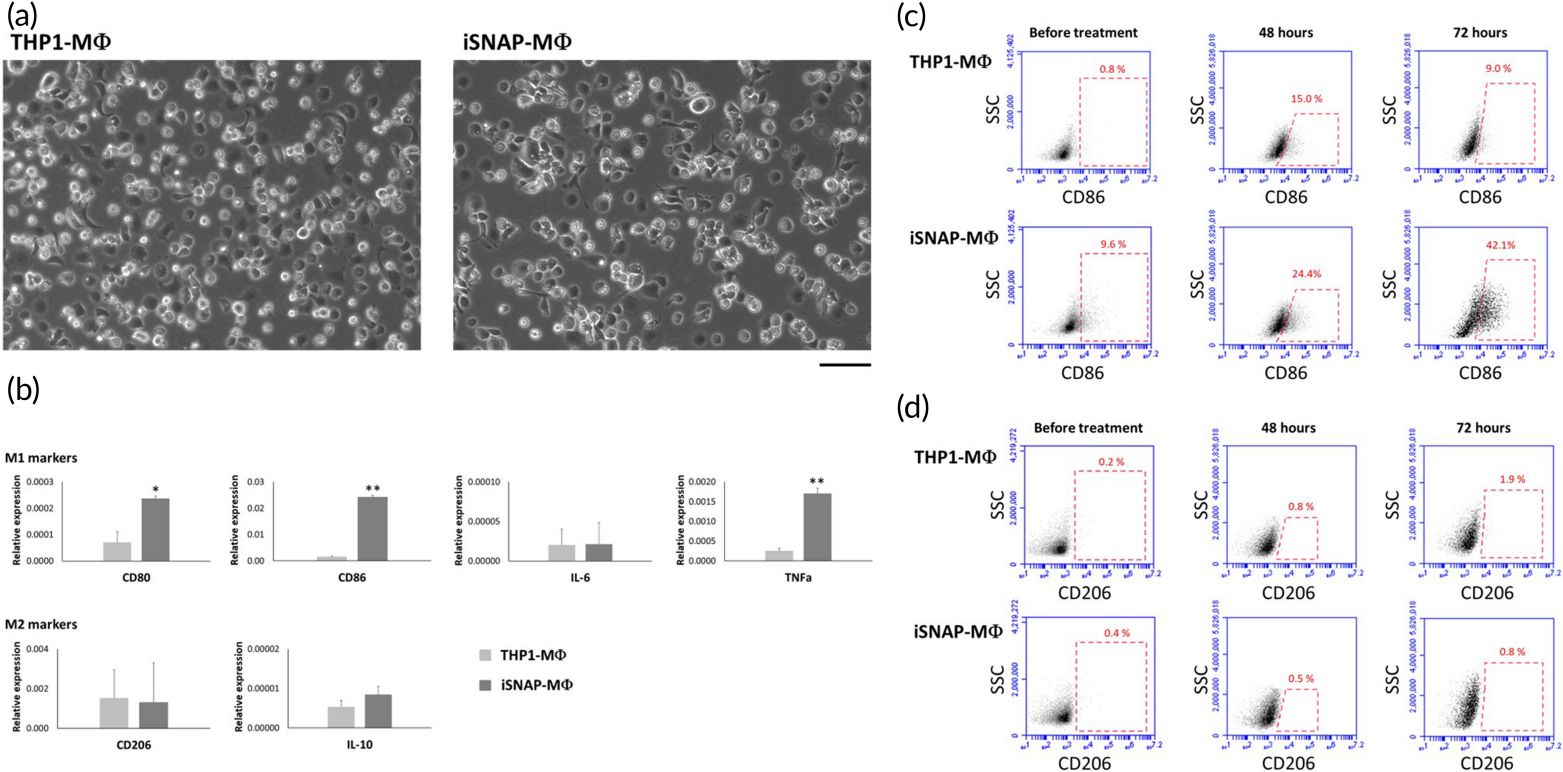
M1 polarization of iSNAP‐MΦ. (a) iSNAP‐MC and THP1 are differentiated to macrophages (iSNAP‐MΦ and THP1‐MΦ) by PMA treatment. (b) mRNA expression levels of M1 and M2 markers in iSNAP‐MΦ and THP1‐MΦ. Percentages of (c) CD86^+^ and (d) CD206^+^ cells in iSNAP‐MΦ and THP1‐MΦ. Scale bar = 50 μm

We then treated iSNAP‐MC and THP1 with PMA to differentiate into macrophages (iSNAP‐MΦ and THP1‐MΦ) and study their phenotypes. Most cells after differentiation showed round shapes in iSNAP‐MΦ and THP1‐MΦ (Figure 3a). The mRNA expression level of markers of M1 MΦ (CD80, CD86, IL‐6, and TNFα) and M2 MΦ (CD206 and IL‐10) were then analyzed in iSNAP‐MΦ. iSNAP‐MΦ showed a significant upregulation of CD80, CD86, and TNFα expressions comparing to the control group of THP1‐MΦ (Figure 3b). The results of surface protein markers of M1 (CD80 and CD86) and M2 (CD206) phenotypes in iSNAP‐MΦ were further confirmed by flow cytometry. The results indicate an increase of M1 CD86^+^, but not M2 CD206^+^ cells in iSNAP‐MΦ (Figure 3c,d). Moreover, there is no significant difference in the expressions of CD206 and IL‐10 between iSNAP‐MΦ and THP1‐MΦ (Figure 3b). These data indicate the polarization of M1‐like phenotype in iSNAP‐MΦ, although the M1 cytokine marker IL‐6 level was not elevated in these cells for reasons currently unclear.

### 3.4 iSNAP‐MΦ have increased secretion of proinflammatory chemokines and cytokines

**FIGURE 4:**
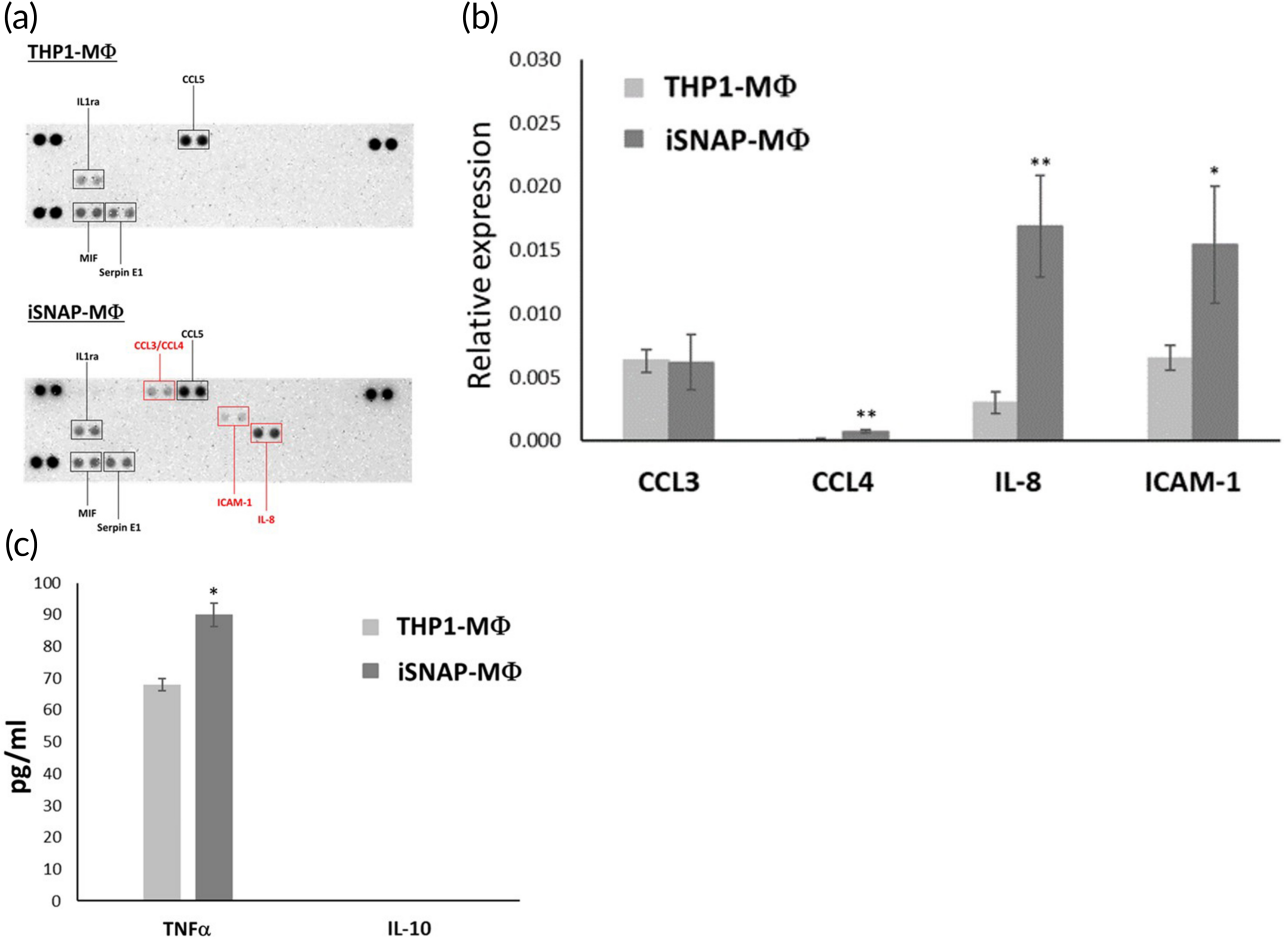
Secretory profile of chemokines and cytokines in iSNAP‐MΦ. (a) Secretory profile in iSNAP‐MΦ and THP1‐MΦ was analyzed by cytokine array. (b) mRNA expressions of CCL4, IL‐8, and ICAM‐1 in iSNAP‐MΦ and THP1‐MΦ were validated by quantitative PCR. (c) TNFα and IL‐10 secretions were analyzed in iSNAP‐MΦ and THP1‐MΦ

Proinflammatory chemokines and cytokines have been reported to increase in M1 macrophages.^19^, ^20^ To investigate the secretion profile of iSNAP‐MΦ, we used a cytokine array to analyze the conditioned medium after induction of macrophage differentiation for 2 days. As shown in Figure 4a, secretions of CCL3/4, ICAM‐1, and IL‐8 increased in iSNAP‐MΦ compared to THP1‐MΦ. Quantitative PCR verified that the mRNA expression levels of CCL4, ICAM‐1 and IL‐8 were upregulated in iSNAP‐MΦ (Figure 4b). TNFα and IL‐10 are widely used as critical markers for function of M1 and M2 macrophages.^20^ Quantification by ELISA shows that TNFα secretion increased significantly in iSNAP‐MΦ (Figure 4c), whereas IL‐10 secretion was nondetectable in both iSNAP‐MΦ and THP1‐MΦ (Figure 4c). These lines of evidence show increases of proinflammatory chemokines and cytokines in iSNAP‐MΦ.

### 3.5 Treatment with iSNAP‐MC inhibit tumor progression of human B‐lymphoma in vivo

**FIGURE 5:**
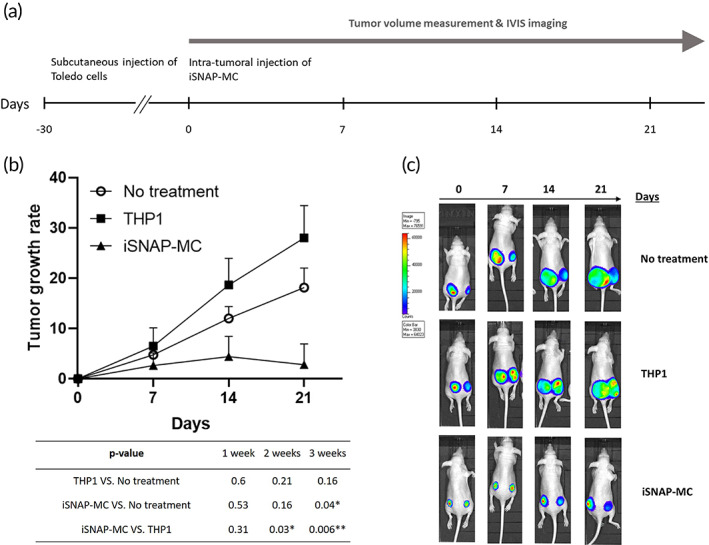
iSNAP‐MC treatment inhibits tumor progression of human B‐lymphoma in vivo. (a) The protocol of treatment plan. Mice were injected with *pHIV‐Luc‐ZsGreen* transfected Toledo cells subcutaneously, and tumor formation was observed after 30 days. The tumors were treated with 1x10^6^ iSNAP‐MC or THP1. Tumor volume was measured by a caliper weekly. (b) The results of tumor volume measurements with statistical analysis. The table shows *p* values for comparison between groups. (c) IVIS imaging in the group of no‐treatment, THP1 and iSNAP‐MC. *N* number: no‐treatment, *n* = 7; THP1, *n* = 4; iSNAP‐MC, *n* = 4

**FIGURE 6:**
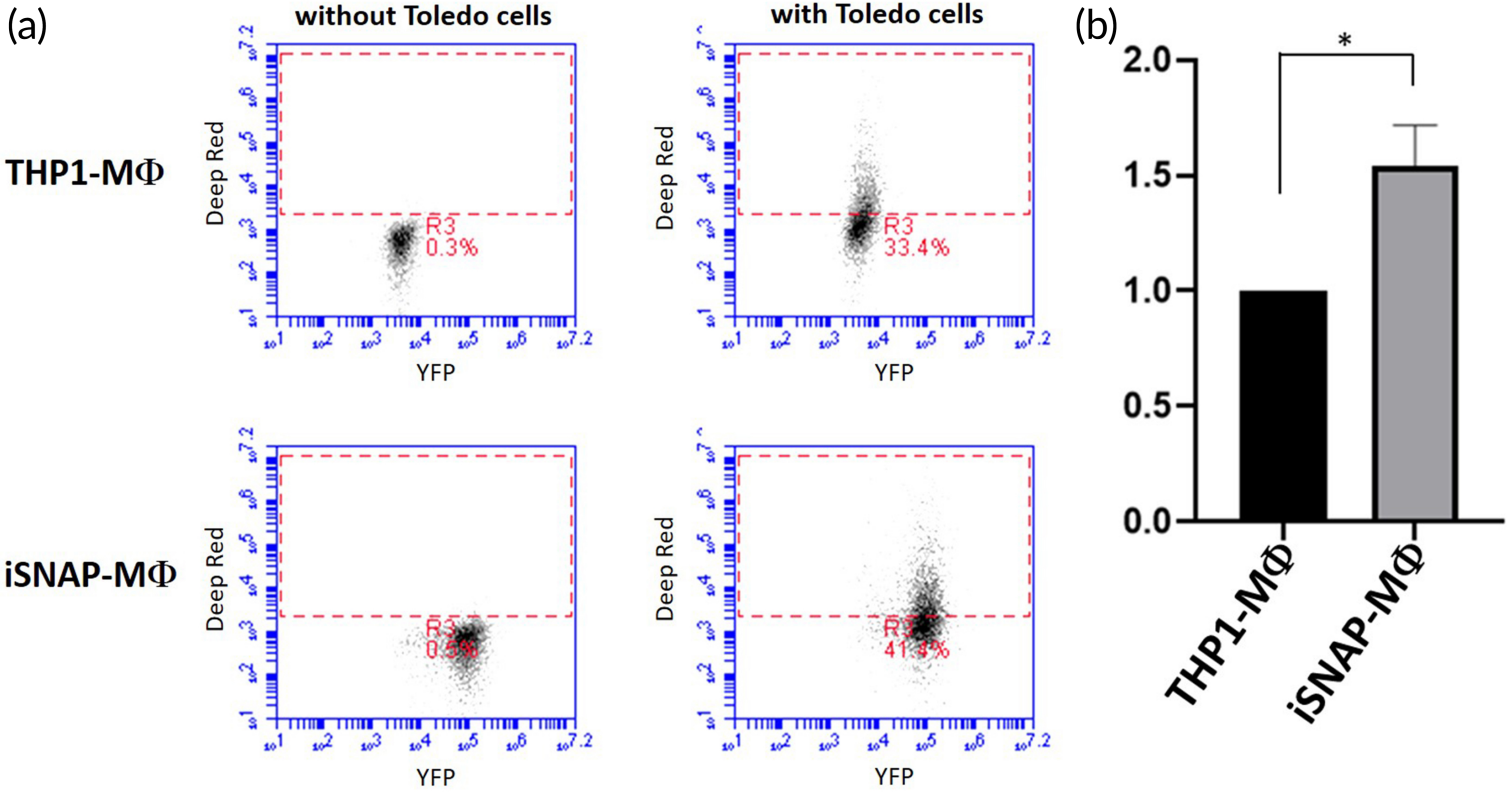
The increase of phagocytic ability in iSNAP‐MC. iSNAP‐MC and THP1 were induced to differentiate into macrophages by PMA treatment and then cocultured for 4 h with Toledo cells labeled by Mito Tracker Deep Red. After PBS washing twice, the cells were trypsinized and resuspended for flow cytometry analysis. (a) Signal of Deep Red was investigated in iSNAP‐MΦ and THP1‐MΦ coculturing with and without labeled Toledo cells. (b) The percentage of Deep Red signal was normalized and quantified as phagocytic index (*n* = 3)

To investigate whether iSNAP‐MC treatment can inhibit tumor progression of human B‐lymphoma, Toledo cells which express high levels of CD47 were transfected with *pHIV‐Luc‐ZsGreen* and injected subcutaneously into mice (Figure S1). Thirty days after the Toledo cell inoculation, when the tumor growth is observed to reach 5 mm in diameter, we started to treat the tumors by intratumoral injection of iSNAP‐MC, confirmed by YFP immunostaining (Figure S2). The protocol of treatment plan is shown in Figure 5a, and the tumor volume was measured at 7, 14, and 21 days by caliper and IVIS imaging. As shown in Figure 5b, the iSNAP‐MC treatment inhibited the tumor growth at Day 21 compared to either THP‐1 treated or no‐treatment group. We further verified that iSNAP‐MC cells indeed showed a clear enhancement of phagocytic ability compared to THP1‐derived macrophages when cocultured with Toledo tumor cells (Figure 6). ELISA experiments showed that inflammatory cytokine IL‐1β was undetectable in the serum of mice under the iSNAP‐MC treatment for 1 week (Figure S3). These results suggested that the injected iSNAP‐MC may suppress tumors via the enhanced phagocytosis, without causing systematic inflammatory responses in the host animals. This is consistent with previous reports showing that intratumoral injection had lower systemic diffusion comparing to intravenous injection,^21^, ^22^ and intratumoral injection has limited systemic inflammation.^23^, ^24^

## 4 DISCUSSION

Tumor cells can escape from human immunity in many ways.^25^ TAMs and tumor microenvironment play important roles in helping tumor cells to prevent attacks from immune cells and the subsequent activation of immune responses.^26^, ^27^ The tumor microenvironment is complex, and the cross‐talk between tumor cells and TAMs can modulate tumor microenvironment and promote tumor progression. In this study, we established a SIRPα‐engineered human monocyte cell line, iSNAP‐MC, with the “Don't eat me” signal rewired. iSNAP‐MC showed an upregulation of monocyte/macrophage markers that reflect a potential enhancement in their phagocytotic ability and cell transmigration. The differentiated iSNAP‐MΦ expressing iSNAP‐M also displayed molecular signals and phenotypes mimicking M1 macrophages. Consistent with this, the secretion of proinflammatory chemokines and cytokines was increased in iSNAP‐MΦ. These lines of evidence reveal a potential mechanism of iSNAP‐MC/MΦ to modulate tumor environment for treatment of cancers.

Engineering monocytes/macrophages is still at its early stage as compared to genetically engineered T cells, particularly chimeric antigen receptor T cells (CAR T cells).^28^ CAR T cells are generated to attack tumor cells directly, while engineered monocyte/macrophages engulf tumor cells, with subsequent antigen presentation leading to T cell activation, or induce Th1 responses via M1 activation. Because monocytes can be purified from peripheral blood with abundant amount and naturally differentiated into macrophages at target tumor sites in vivo,^29^ we reengineered monocytes with the rewired genetic modules and introduced them into a tumor mice model to examine the immunotherapy efficiency in vivo. The M1 polarization in iSNAP‐MΦ in vitro and the repression of human B‐lymphoma by iSNAP‐MC treatment in vivo (Figures 2, 3, 4, 5) indicate a potential therapeutic strategy via reengineering monocytes/macrophages to rewire signaling pathways for cellular activations.

In solid tumors and leukemia, there are many ongoing clinical trials of genetically engineered T cells.^30^ While intravenous injection is a conventional route in clinics, the efficiency of trafficking to tumors is an important issue for solid tumors. Although we have not tested directly in the current study, the engineered monocytes, with their trafficking capability, can be intravenously injected to target tumors that can not be reached by local injection. Our approach using the engineered monocytes can hence be readily extended to different types of cancers where CD47 is highly expressed, including acute leukemia, NHL, colorectal, and ovarian cancers.^31^ In fact, SIRPα‐blocked macrophages primed with tumor targeting antibody have been reported to traffic to solid tumor after intravenous injection.^6^ Accordingly, intravenous injection of iSNAP‐MC will be further tested to investigate the efficiency of trafficking to tumors and efficacy of tumor repressing in the future.

Figure 2c shows that the mRNA expression of endogenous SIRPα was also observed in iSNAP‐MΦ; although this is much lower than that of the engineered SIRPα, endogenous SIRPα should still have functional activity to result in the partial blockade of the “Don't eat me” signal. A knockout of endogenous SIRPα by genetic engineering should hence further improve the efficiency of our approach. As shown in Figures 3 and 4, iSNAP‐MΦ exhibit the phenotype of M1 macrophages, with increased secretion of proinflammatory chemokines and cytokines. With next‐generation sequencing and proteomics technology, the downstream molecular candidates of iSNAP can be further identified and modulated to improve the therapeutic efficacy of iSNAP‐MC. Furthermore, in the present study, a single dose of iSNAP‐MC by intratumoral injection was used to treat tumor. While this iSNAP‐MC treatment can inhibit tumor progression of human B‐lymphoma (Figure 5), varying the dosage and duration of iSNAP‐MC may further enhance the therapeutic efficacy. Earlier studies indicated that coculture with tumors cells could induce the polarization of THP1 or THP1‐derived macrophages toward M2 phenotype.^32^, ^33^, ^34^, ^35^ Furthermore, THP‐1‐derived TAMs were reported to show M2 polarization and promote tumor growth.^36^ The injected THP‐1 cells in our work also promoted the tumor growth, possibly through a similar mechanism. It will hence be interesting in the future to investigate the effect of injected monocytes on the surrounding TAMs in inhibiting tumors, on top of the phagocytic action engulfing tumor cells.

While nanoparticles can be administered to inhibit CSF1R and SHP2 signaling to enhance the phagocytic ability of macrophages in vivo, the materials of these nanoparticles, for example, phosphatidyl choline, have not been approved by FDA for intratumoral or subcutaneous injections.^37^ In contrast, cell‐based therapy utilizing immune cells, including macrophages, T cells, and natural killer cells, have been well‐established for clinical applications with long term effects.^38^, ^39^, ^40^ As such, compared to nanoparticle‐based drug delivery systems targeting macrophages, genetically engineered cells, including macrophages, are more biocompatible with clinical practices and have longer‐term effect. Genetics can be further designed to enhance the tumor infiltration and antitumor cytokine release of these engineered cells. Therefore, genetically engineered macrophages/monocytes should have tremendous potentials to be translational toward clinical medicine in the future.

Antibody‐dependent cellular phagocytosis of macrophages has been reported to be involved in cancer immunity.^41^ In this study, we have demonstrated that iSNAP‐MC treatment can inhibit tumor progression of human B‐lymphoma, it would be warranted to conduct further investigations to examine whether iSNAP‐MC treatment can inhibit progression in other kinds of tumors which do not respond to antibody drugs and do not have specific markers to target. In fact, the engineering of macrophages is a rapidly advancing field for cancer immunotherapy.^42^ In clinical trials, blockage of CD47‐SIRPα recognition with antibody drugs has also been reported and is under ongoing testing.^43^ In clinical settings, CAR T‐cell therapy with T‐cytotoxic cells expressing chimeric antigen receptors has shown efficacy in cancer treatment.^44^ In this study, we established engineered human monocytes via overexpression of engineered‐SIRPα to rewire the “Don't eat me” signal. As such, our findings pave a solid ground for applying reengineered monocytes or macrophages toward cancer immunotherapy.

## 5 CONCLUSION

**FIGURE 7:**
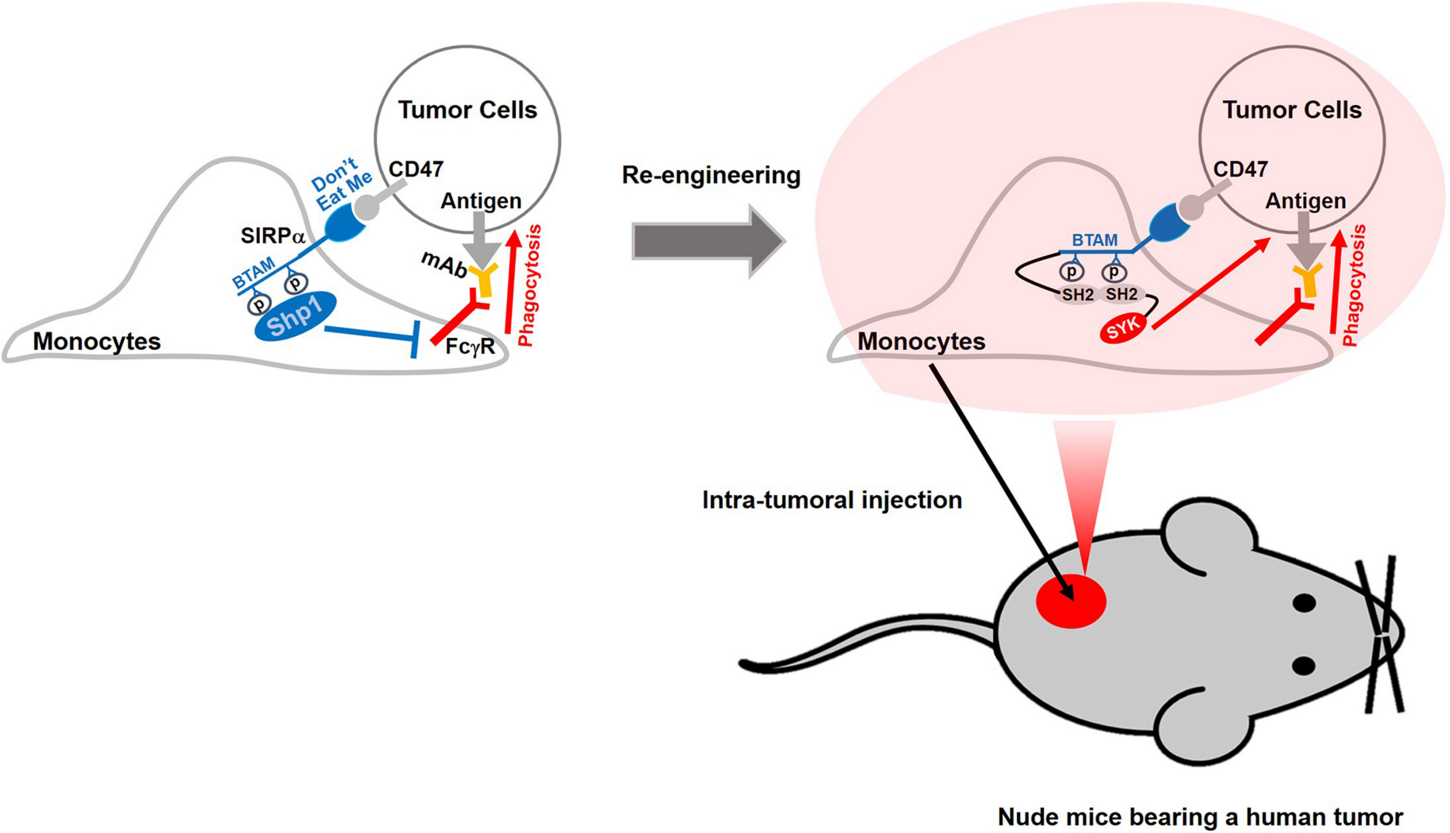
Scheme of iSNAP‐MC to inhibit tumor growth. Re‐engineered monocytes with integrated sensing and actuating proteins (iSNAPs) rewire the repressive CD47‐SIRPα axis into activation signaling pathways and inhibit tumor progress

In this study, we have reengineered monocytes with integrated sensing and actuating proteins (iSNAPs) to rewire the repressive CD47‐SIRPα axis into activation signaling pathways. Our results show that the reengineering monocytes exhibit phenotype of M1 polarization after induction of macrophage differentiation. Also, injection of the reengineered monocytes into mice bearing human B‐lymphoma tumors led to the suppression of tumor progression (Figure 7). As such, the integration of synthetic biology and immune engineering can be powerful for the translational applications in cancer immunotherapy.

## AUTHOR CONTRIBUTIONS

**HaoHsiang Wu:** Investigation (equal); methodology (equal); validation (equal); writing – original draft (equal). **Siamak Amirfakhri:** Formal analysis (equal); investigation (equal); methodology (equal). **Hannah Hollandsworth:** Investigation (equal); methodology (equal). **Filemoni Filemoni:** Investigation (equal); methodology (equal). **Yahan Liu:** Investigation (equal); methodology (equal). **Yiqian Wu:** Investigation (equal); methodology (equal). **Hsin‐Hung Lin:** Investigation (equal); methodology (equal). **Julie Y.S. Li:** Writing – review and editing (equal). **Hongquan Xu:** Formal analysis (equal); methodology (equal). **Shu Chien:** Conceptualization (equal); writing – review and editing (equal). **Michael Bouvet:** Conceptualization (equal); writing – review and editing (equal). **Yingxiao Wang:** Conceptualization (equal); writing – review and editing (equal).

## CONFLICT OF INTERESTS

Yingxiao Wang is a scientific cofounder of Cell E&G and Acoustic Cell Therapy, Inc. Other authors declare no conflict of interests.

### PEER REVIEW

The peer review history for this article is available at https://publons.com/publon/10.1002/btm2.10285.

## Supporting information


**Appendix** S1: Supporting Information

**Table S1** Primer list.

## Data Availability

The data that support the findings of this study are available from the corresponding author upon reasonable request.
