# Supplementary figures and images for: Monocytes engineered with iSNAP inhibit human B‐lymphoma progression
Source: Bioeng Transl Med. 2022 Jan 12;7(2):e10285. doi: 10.1002/btm2.10285 (PMC9115687; doi:10.1002/btm2.10285)

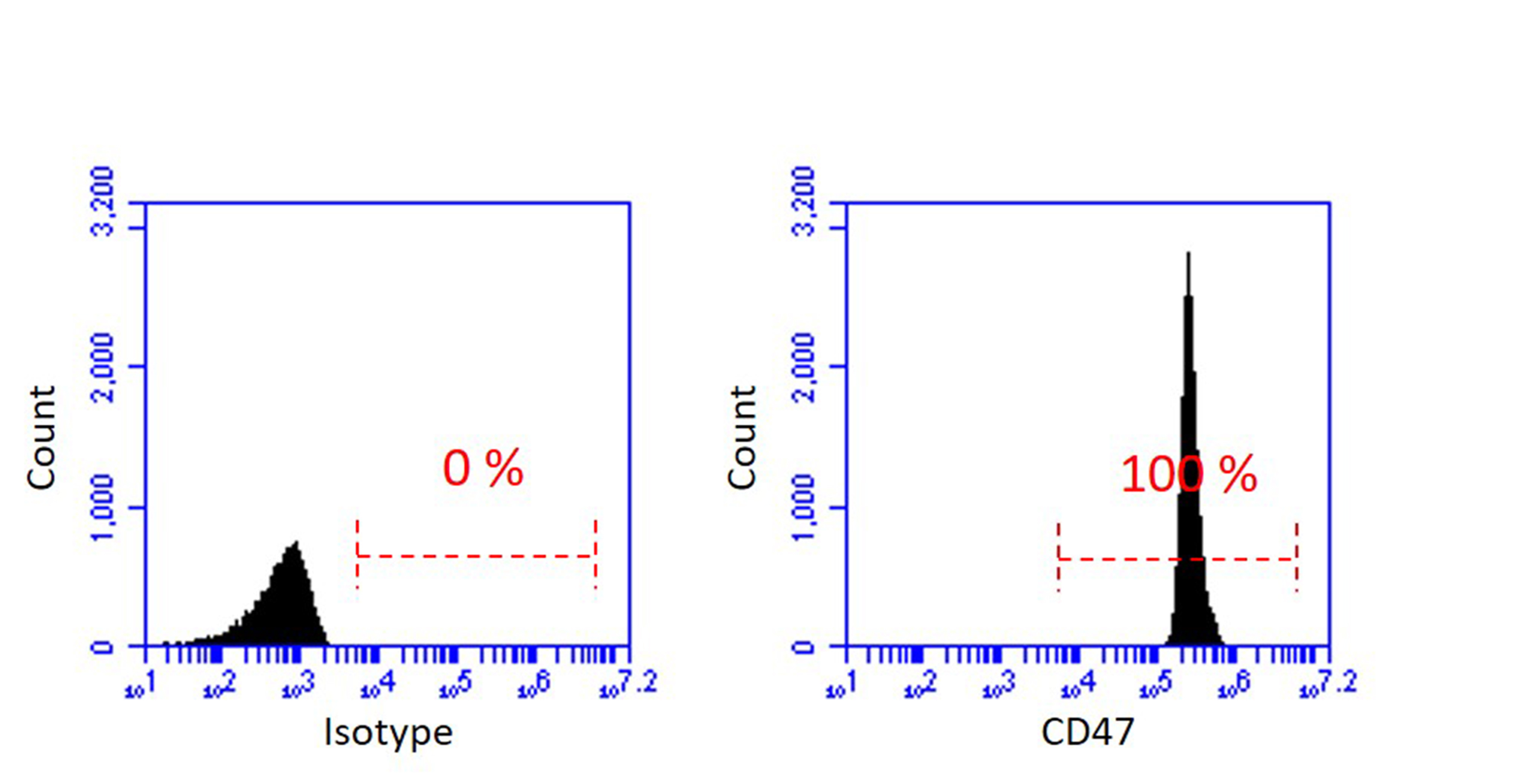

Supplement: Supplementary file 1 — Appendix S1: Supporting Information [file BTM2-7-e10285-s001.jpg]
