# Supplementary material for: Monocytes engineered with iSNAP inhibit human B‐lymphoma progression
Source: Bioeng Transl Med. 2022 Jan 12;7(2):e10285. doi: 10.1002/btm2.10285 (PMC9115687; doi:10.1002/btm2.10285)
Supplement: Supplementary file 2 — Table S1 Primer list. [file BTM2-7-e10285-s002.docx]

**Table 1. Primer list.**

| **Target** | **Sequence** |
| --- | --- |
| Human CD11b | Forward: AGGACTCTGCCCAGACCAC  Reverse: GTCGGGGATACTTCGCTGT |
| Human CD14 | Forward: GAATGGAGACGCCCACAG  Reverse: TCGGAGCGCTAGGGTTTAC |
| Human CD31 | Forward: GGTGGATGAGGTCCAGATTTC  Reverse: TTGCAGCACAATGTCCTCTC |
| Human CD80 | Forward: GAAGCAAGGGGCTGAAAAG  Reverse: GGAAGTTCCCAGAAGAGGTCA |
| Human CD86 | Forward: CAGAAGCAGCCAAAATGGAT  Reverse: GAATCTTCAGAGGAGCAGCAC |
| Human CD206 | Forward: ACACCAAAACCTGAGCCAAC  Reverse: CCACCCATCTTCAGTAACTGGT |
| Human TNFα | Forward: AGCCCATGTTGTAGCAAACC  Reverse: TCTCAGCTCCACGCCATT |
| Human IL-6 | Forward: GATGAGTACAAAAGTCCTGATCCA  Reverse: CTGCAGCCACTGGTTCTGT |
| Human IL-10 | Forward: TGCCTTCAGCAGAGTGAAGA  Reverse: GCAACCCAGGTAACCCTTAAA |
| Human CCL3 | Forward: CAGAATCATGCAGGTCTCCAC  Reverse: GCGTGTCAGCAGCAAGTG |
| Human CCL4 | Forward: CTGCTCTCCAGCGCTCTC  Reverse: ACCACAAAGTTGCGAGGAAG |
| Human IL-8 | Forward: AGACAGCAGAGCACACAAGC  Reverse: ATGGTTCCTTCCGGTGGT |
| Human ICAM-1 | Forward: CCTTCCTCACCGTGTACTGG  Reverse: AGCGTAGGGTAAGGTTCTTGC |
| Endo-SIRPα | Forward: GCCTGGGGTCCAAGAACT  Reverse: GCAGTCGAGGGTCTTCAAAA |
| Engineered-SIRPα | Forward: TGGTGGTGTTCAATTAGCTGAC  Reverse: TGATAGGATAAGTAATGGTTGTCTGG |
| Human GAPDH | Forward: ATGACATCAAGAAGGTGGTG  Reverse: CATACCAGGAAATGAGCTTG |
